# Doctors and Death as a Natural Instinct

**Published:** 1907-01-26

**Authors:** 


### The Hospital

A Journal of

The Medical Sciences and Hospital Administration.

Vol. XLI.—No. 1,063. SATURDAY, JANUARY 26, 1907.

### Doctors and Death as a Natural Instinct.

"How wonderful is Death—
Death and his brother Sleep."
Queen Mab.

The gift of poetic imagination is rarely associated with the thoroughness needed by those who advance science by investigation. But when the two faculties are combined, they prove to be of singular value to the individual, and, through him, to the world of science. Professor Metchnikoff is so endowed, and he has recently laid before the readers of *Harper's Magazine* an interesting contribution on the causes of natural death—that is to say, death from old age as opposed to death from disease or accident. He discusses it from the broad standpoint of the accomplished biologist. On the assumption that sleep is the result of an auto-intoxication, associated with the presence of an excess of lactic acid in the system, he submits that it is probable that "death is simply due to poisoning by the harmful elements generated by the activity of our organs." Sleep, he thinks, is an instinctive need for rest; natural death in like manner is the manifestation of an instinctive want, and the instinct of death is often seen in very old people, who die as easily and quietly as children fall asleep. This instinct of death has always been recognised, and it has been associated from all time with the gift of prophecy, for, as Aretæus tells us in his essay on the burning fever of Hippocrates, "the soul, whilst shuffling off this mortal coil and disentangling itself from the incumbrances of the body, becomes purer, more essential, more entirely spiritual, as if it had already commenced its new existence"; or, as Shelley phrased it, "like an unbodied joy whose race is just begun." The predictions of Jacob before "he drew up his feet into the bed and yielded up the ghost" are known to everyone, and the poets have made full use of this gift of prophecy just before death. Sophocles in the "Œdipus Coloneus" represents Œdipus, as he is walking to the spot where he is to die, foretelling to Theseus the prosperity of Athens and of his family. Virgil makes Orodes prophesy the death of Mezentius. Old John of Gaunt says as he dies, "Methinks I am a prophet new inspired!"

But medical men are concerned more with the practical side of death, than with the folk-lore and superstitions which have gathered round it. It is their duty to attend the death-bed and to alleviate sufferings associated with it, as far as possible, making the departure from this life easy and gentle. This is a part of their art to which Francis Bacon in his treatise "De Augmentis Scientiarum" applied the term "Euthanasia." The subject deserves the most careful study, for it well repays anyone who has made himself master of it. No broad principles are involved; it consists wholly of details of isolated acts of attention done at the precise moment when they are needed. The restlessness of the dying, for instance, is often relieved by lightening the weight of the bedclothes, or by opening a window to diminish the heat of the room. The sinking and exhaustion, so characteristic of a failing heart, are best relieved by milk, cream, and eggs beaten up, rather than by more solid food which the patient takes under protest, or even beef-tea and extracts of meat. If stimulants are given, they should be administered in small quantities at a time, and repeated at such short intervals that the effects upon the heart and pulse become continuous. Of wines, it is thought that Madeira, from its slight acidity, is specially agreeable to the palate, whilst it is both sustaining and cordial; but Tokay is often more acceptable, especially to those who are sinking from such exhausting diseases as hæmorrhage and prolonged suppuration. Champagne is often eagerly taken, but its effects soon pass off, and it needs to be repeated at shorter intervals than other wines. The dry and parched condition of the throat, so common in the dying, is often improved by weak black tea, without milk and slightly acidulated with a slice of lemon.

It is told traditionally that John Hunter, who once had the chief surgical practice in London, used often to exclaim, "Thank God for opium!" but opium in all its forms must be used with discretion, not as an anodyne to relieve pain and mask symptoms, rather as a cordial and carminative to allay the sinking and anguish about the heart and stomach, which are common in the dying. It is one of the tests of him, who has mastered the art of making a deathbed bearable, that he knows when and in what doses to use laudanum or morphine, and when to refrain from their use. For in the beautiful prose of De Quincey, "Simultaneously with the conflict, the pain of conflict has departed, and thenceforward the gentle process of collapsing life, no longer fretted by counter-movements, slips away with holy peace, into the noiseless deeps of the Infinite."

